# Effect of Radial Stress on the Nanoparticle-Based Electrolyte Layer in a Center-Wound Roll with Roll-to-Roll Systems

**DOI:** 10.3390/nano12061014

**Published:** 2022-03-20

**Authors:** Jaehyun Noh, Minho Jo, Gyoujin Cho, Sanghoon Nam, Changwoo Lee

**Affiliations:** 1 Department of Mechanical Design and Production Engineering, Konkuk University, 120 Neungdong-ro, Gwangjin-gu, Seoul 05029, Korea; zenty616@konkuk.ac.kr (J.N.); als8080@konkuk.ac.kr (M.J.); 2 Department of Biophysics, Institute of Quantum Biophysics, Sungkyunkwan University, Suwon 16419, Korea; gcho1004@skku.edu; 3 Department of Mechanical Engineering, Massachusetts Institute of Technology, Cambridge, MA 02139, USA; shnam@mit.edu; 4 Department of Mechanical Engineering, Konkuk University, 120 Neungdong-ro, Gwangjin-gu, Seoul 05029, Korea

**Keywords:** coated layer defects, electrolyte, radial stress, residual stress, roll-to-roll slot-die coating, taper tension profile, winding process

## Abstract

Recently, slot-die coating based on the roll-to-roll process has been actively used to fabricate nanoparticle-based electrolyte layers because it is advantageous for high-speed processes and mass production of uniformly thick electrolyte layers. In this process, the fabricated electrolyte layer is stored as a wound roll throughout the rewinding process. We analyzed the defects and geometric changes in an electrolyte layer, i.e., gadolinium-doped cerium oxide (GDC), due to the radial stress in the wound roll. We found that the thickness of the coated layer could be decreased by increasing the radial stress, i.e., cracks can be generated in the coated layer if excessively high radial stress is applied to the wound-coated layer. More thickness changes and crack defects were generated with time due to the residual stress in the wound roll. Finally, we analyzed the effects of taper tension profiles on the defects of the coated layer in the wound roll and determined the taper tension profile to minimize defects.

## 1. Introduction

Roll-to-roll systems are suitable candidates for achieving high speed and mass production of flexible functional devices, such as flexible printed electronics, solar cells, fuel cells, bioelectric sensors, and pressure capacitive sensors [1,2,3,4,5,6,7]. The slot-die coating process has the advantages of a large-area and pre-metered coating; therefore, it is used in the mass production of large-area functional layers [8,9,10,11]. Roll-to-roll slot-die coating systems consist of unwinding, infeeding, coating, drying, outfeeding, and rewinding sections [12,13]. The coated layer, manufactured through a drying process after coating, is stored in a wound roll throughout the rewinding process, which is advantageous as a large amount of coated products can be stored compactly. In this process, stress in the radial direction, called radial stress, and stress along the roll circumference, called hoop stress, are applied to the wound roll [14,15].

Several mathematical models have been developed to analyze the behavior of internal stresses in a wound roll. Altmann derived a general solution for predicting interface pressure and tensile stress inside a wound roll. Thereafter, various studies were published to explain the internal stress of the wound roll based on his model [16]. Yagoda obtained an exploratory closed-form analytical solution for radial and hoop stresses and presented the behavior of internal stresses around the core. He studied the behavior of internal stresses around the core of a wound roll and presented a guideline for designing the core to prevent roll collapse [17]. Unlike previous studies, Hakiel introduced nonlinear material properties to derive a nonlinear orthotropic hoop stress model in the center-wound roll. In addition, Hakiel was the first to propose a numerical approximation approach, in which the stress in a wound roll was predicted by introducing a radial-direction modulus that changes with the interlayer pressure [18]. Burns further explained the elastic stress of the wound roll with consideration to the residual strain, along with the elastic strain of the web. He suggested that stresses applied to the web created residual strains in the wound roll. In his study, residual strains in the wound roll were relaxed and equilibrated, such that residual stresses affected the magnitude of radial and hoop stresses [19]. Lee et al. confirmed that a previously developed radial stress model can only be applied to web materials with low density and bending strength. Accordingly, he developed a model based on Burns’ model that included the density, bending stress, and residual stress of web materials. In his model, the characteristics of web materials could be confirmed by the influence of the gravitational effect and residual stress within the radial stress [20].

In the process of simultaneously manufacturing roll products with large-area materials at a higher speed than before in industrial sites, more materials with a large maximum radius ratio are being wound to increase productivity. As a result, high radial stress occurs inside the wound roll, resulting in frequent defects due to internal stresses in the roll-to-roll system-based process. Accordingly, research has been conducted to predict the range of radial stress to prevent defects; however, only a few studies have analyzed the effects of the radial stresses of center-wound rolls on defects in a coated layer. If the radial stress in the wound roll is excessively high, then the wound coated layer may be deformed or cracked. Therefore, it is essential to analyze the effects of internal stresses on the quality of the functional layer and determine the radial stress range to prevent defects in the wound roll [21]. In this study, we analyzed the impact of radial stress, which increases over time due to residual stress, on the quality of the coated layer inside a wound roll manufactured through a roll-to-roll slot-die coating system. We coated a gadolinium-doped cerium oxide (Sigma Aldrich, St. Louis, MO, USA) layer, an electrolyte layer of a solid oxide fuel cell (SOFC), on a plastic web (CD901, KOLON Inc., Seoul, Korea), using a roll-to-roll slot-die coating process, and wound the layer through the rewinding process [22,23,24]. The stabilized zirconia-based electrolyte layer, which has been widely used in the production of SOFC, has to be operated at a temperature of 900–1000 °C or higher to ensure sufficient ionic conductivity. Various studies have been conducted on manufacturing SOFCs with low operating temperatures as they have great advantages of extending the life of the stack and reducing production costs. Gadolinium-doped ceria-based electrolytes are attracting attention as new SOFC electrolyte layers as they can ensure high ionic conductivity at an operating temperature of about 700 °C [25,26].

To compare the degree of defects in the coated layer, depending on the winding tension profiles, a constant tension profile, a linear taper tension profile, and a parabolic taper tension profile were applied. The winding tension is a major influence in the rewinding process that determines the radial stress value inside the wound roll. Therefore, we predicted the radial stresses that depend on the winding tension profile using Burns’ radial stress model. A force-sensing resistor (FSR, FlexiForce B201, Tekscan Inc., Boston, MA, USA) was employed to observe the changes in radial stress due to residual stress in the wound roll, according to the wound roll storage time. After the radial stress measurement over time, coated layer samples for the roll right after the winding process and for the roll with 16 h of aging after the winding process were made. Accordingly, the effect of radial stress on the electrolyte layer was analyzed while observing the defects, geometrical changes, and electrical performances of the coated layer appearing at radius ratios of 1.10, 1.35, 1.85, and 2.45.

## 2. Winding Tension and Radial Stress

**Figure 1:**
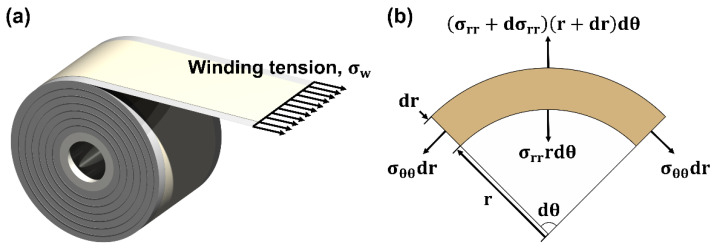
(**a**) Schematic of the winding process. (**b**) Forces in an element of a wound roll.

Figure 1 shows a schematic diagram of the winding tension applied to the web during the wound coated layer manufacturing process. During the winding process, as the radius ratio of the wound roll increases, the amount of wound material increases, so the radial stress in the inner layer increases. Excessive radial stress in the wound roll may cause defects, such as local deformation, starring defects, and telescoping, which may degrade the quality of wound roll products [21]. Accordingly, a method for optimizing a taper tension profile to manufacture a high-quality wound roll has been actively studied, and a linear taper tension profile (σ_w-L_), a hyperbolic taper tension profile (σ_w-H_), and a parabolic taper tension profile (σ_w-P_) have been proposed [20]. The linear taper tension profile shows a constant tension reduction rate and is applied when the maximum radius ratio is small. The hyperbolic taper tension profile shows a profile in which the tension decreases exponentially with an increase in the radius ratio. However, in the hyperbolic taper tension profile, the reduction of the winding tension at the maximum radius ratio cannot reach the set value if the maximum radius ratio is below 5. The parabolic taper profile is a winding tension model that improves upon the limitations of the hyperbolic taper profile and has the advantage of reducing the tension by the set taper value at any maximum radius ratio. The parabolic taper tension profile can effectively mitigate the radial and residual stresses inside the wound roll. The linear, hyperbolic, and parabolic taper tension profiles can be expressed as Equations (1)–(3), respectively.

(1) $$\sigma_{w\text{-}L}\left(r\right)=\sigma_{0}\left[1-\left(\frac{\mathrm{taper}}{100}\right)\cdot \frac{\left(r-1\right)}{\left(R-1\right)}\right],$$

(2) $$\sigma_{w\text{-}H}\left(r\right)=\sigma_{0}\left[1-\left(\frac{\mathrm{taper}}{100}\right)\cdot \left(\frac{r-1}{r}\right)\right],$$

(3) $$\sigma_{w\text{-}P}\left(r\right)=\sigma_{0}\left[1-\left(\frac{\mathrm{taper}}{100}\right)\cdot \frac{1}{\left(R-1\right)}\sqrt{\left(r-1\right)\left(2R-r-1\right)}\right],$$

where r is radius ratio and R is maximum radius ratio.

Burns et al. derived a mathematical model expressing internal stresses within a center-wound roll. Based on the force balance equation, the correlation between the hoop stress and radial stress was derived. The model considers that the residual stress significantly increases the internal stresses of the wound roll. By defining the total strain in the radial direction, as shown in Equation (4), and the total strain in the hoop direction, as shown in Equation (5), the correlation between the radial and hoop strains can be derived using Equation (6).

(4) $$\varepsilon_{\mathrm{rr},\mathrm{total}}=\varepsilon_{\mathrm{rr}}+\varepsilon_{\mathrm{rr}}^{*}=\frac{\mathrm{du}}{\mathrm{dr}},$$

(5) $$\varepsilon_{\theta \theta ,\mathrm{total}}=\varepsilon_{\theta \theta }+\varepsilon_{\theta \theta }^{*}=\frac{\mathrm{ud}\theta }{\mathrm{rd}\theta }=\frac{u}{r},$$

(6) $$r\frac{{d\varepsilon }_{\theta \theta }}{\mathrm{dr}}+\varepsilon_{\theta \theta }-\varepsilon_{\mathrm{rr}}=\varepsilon_{\mathrm{rr}}^{*}-r\frac{{d\varepsilon }_{\theta \theta }^{*}}{\mathrm{dr}}-\varepsilon_{\theta \theta }^{*}.$$

The force balance equation can be applied to the element, as shown in Figure 1b and expressed in Equation (7).

(7) $$-\sigma_{\mathrm{rr}}-r\frac{{d\sigma }_{\mathrm{rr}}}{\mathrm{dr}}+\sigma_{\theta \theta }=0.$$

Equation (7) can be expressed using Equation (8) by defining operator D ($D\equiv r\frac{d}{\mathrm{dr}}$):

(8) $$\left(1+D\right)\sigma_{\mathrm{rr}}=\sigma_{\theta \theta }.$$

By substituting Equations (6) and (8) into the strain and stress terms of the generalized Hooke’s law, respectively, the second-order differential equation, shown in Equation (9), can be obtained as

(9) $$\left\{D^{2}+2D+\left(1-\beta^{2}\right)\right\}\sigma_{\mathrm{rr}}=\sigma^{*},$$

where

(10) $$\beta^{2}=\frac{s_{11}s_{33}-s_{13}{}^{2}}{s_{22}s_{33}-s_{23}{}^{2}}=\frac{s_{11}}{s_{22}},$$

and the residual stress $\sigma^{*}$ is

(11) $$\sigma^{*}=\frac{d}{\mathrm{dr}}\left({r\sigma }_{w}\right)+{\nu \sigma }_{w},$$

The radial stress can be derived by obtaining $\sigma_{\mathrm{rr}}$ in Equation (9):

(12) $$\sigma_{\mathrm{rr}}\left(r\right)=\frac{1}{r}\left[B\left(r^{\beta }-\frac{R^{2\beta }}{r^{\beta }}\right)+\frac{1}{2\beta }\left\{r^{-\beta }\int_{r}^{R}t^{\beta }\sigma^{*}\left(t\right)\mathrm{dt}-r^{\beta }\int_{r}^{R}t^{-\beta }\sigma^{*}\left(t\right)\mathrm{dt}\right\}\right],$$

where

(13) $$B=\frac{2{\beta \sigma }_{0}E_{c}S_{22}-\left[\left\{E_{c}\left(S_{12}-{\beta S}_{22}\right)-1\right\}\int_{1}^{R}t^{\beta }\sigma^{*}\left(t\right)\mathrm{dt}\right]+\left[\left\{1-E_{c}\left(S_{12}+{\beta S}_{22}\right)\right\}\int_{1}^{R}t^{-\beta }\sigma^{*}\left(t\right)\mathrm{dt}\right]}{2\beta \left\{\left(S_{12}E_{c}-1\right)\left(1-R^{2\beta }\right)+{\beta E}_{c}S_{22}\left(1+R^{2\beta }\right)\right\}}.$$

In this study, the winding process was performed using a constant tension profile, a linear taper tension profile represented by Equation (1), and a parabolic taper tension profile represented by Equation (3). The radial stress inside the wound roll was predicted using Equation (12), and the radial and residual stress distribution vary depending on the applied winding tension profile. The magnitude of the radial stress is large in the inner part of the wound roll.

## 3. Experiments

**Figure 2:**
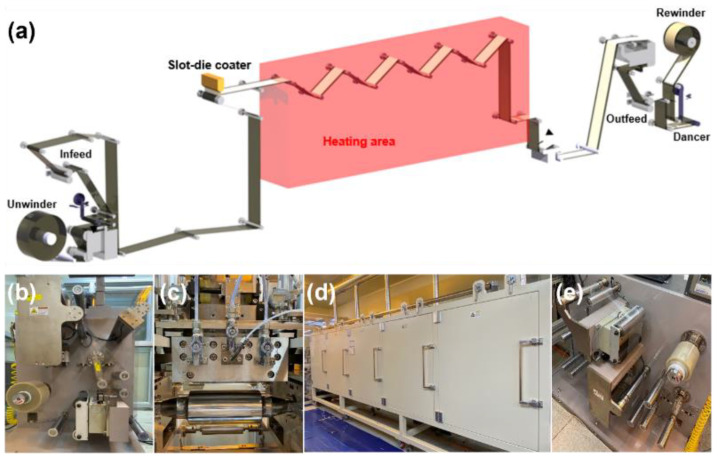
Roll-to-roll manufacturing systems: (**a**) schematic of roll-to-roll slot-die coating systems; (**b**) unwinding and infeeding section; (**c**) slot-die coater; (**d**) dryer; and (**e**) outfeeding and rewinding section.

**Figure 3:**
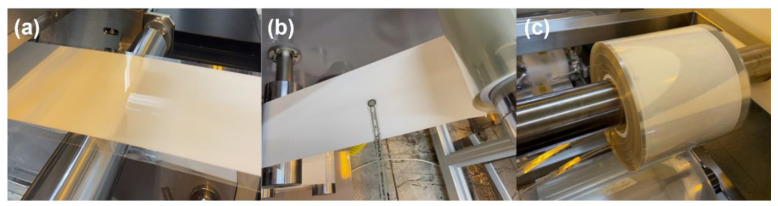
(**a**) Gadolinium-doped ceria coating layer; (**b**) the force-sensing resistor at the rewinding section; and (**c**) wound coated layer aging procedure.

We fabricated a nanoparticle-based gadolinium-doped ceria (GDC)-coated layer with a roll-to-roll slot-die coating machine, as shown in Figure 2a, to investigate the effect of the radial stress, regarding the taper tension profile, on the quality of the coated layer. Figure 2b–e shows the roll-to-roll slot-die coating system installed at Konkuk University. The constant tension, linear taper tension, and parabolic taper tension profiles were used. The taper values of the linear and parabolic taper tension profiles were 20%. Table 1 lists the properties of the web material, GDC ink, and GDC-coated layer used in this study. The GDC-coated layer shown in Figure 3a was wound in the rewinding process and stored for 16 h to identify the effects of internal stresses on the wound roll and the aging time on the quality of the wound GDC layer. FSRs were used to measure the radial stress in the wound roll according to the radius ratio. The maximum force of the FSR was 4448 N. The FSRs were inserted into the wound GDC roll during the winding process when the radius ratios were 1.10, 1.35, and 1.85, as shown in Figure 3b,c.

Table 2 lists the six cases of experimental conditions to identify the effects of radial stress on the GDC coated layer. The initial winding tension was determined by considering the web width, typical tension unit, and pound per linear inch, as proposed in a previous study [27]. Considering the total amount of web materials and estimated range of radial stress, the maximum radius ratio was 2.5. The maximum aging time was 16 h. The measurement interval of the radial stress was 30 min. The surface and cross-section of the GDC layers were measured before and after aging.

**Figure 4:**
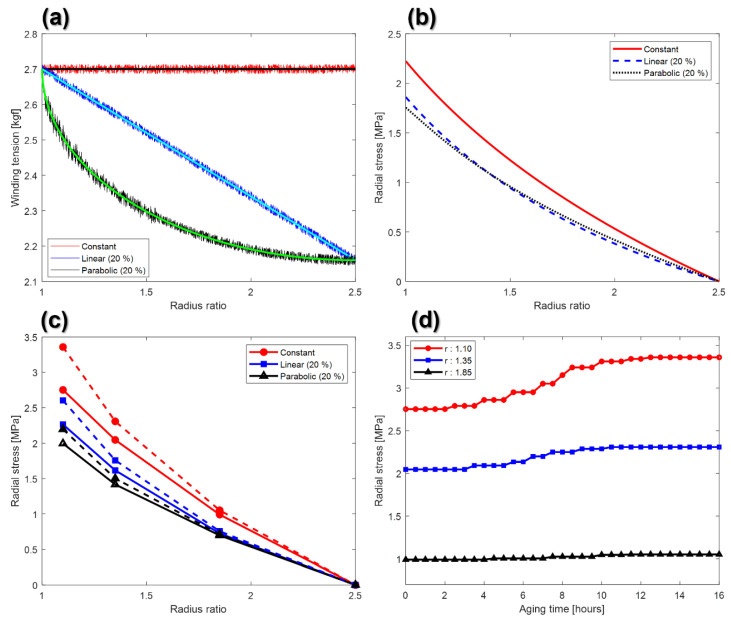
(**a**) Winding tension profile (constant, linear, and parabolic); (**b**) estimated radial stress; (**c**) the measured radial stress with respect to the radius ratio; and (**d**) the measured radial stress for case 2 with respect to the aging time.

Figure 4a presents the setting tension and measured tension according to the winding tension profile. The measured winding tensions were obtained from the load cell and the average value of the tension data for cases, of which the same winding tension profile was applied, was calculated. The maximum estimation errors of the constant tension, linear taper tension, and parabolic taper tension profiles were 0.50%, 0.63%, and 1.46%, respectively. Figure 4b presents the estimated radial stress using Burns’ radial stress model, depending on the taper tension profile. The bold and dotted lines in Figure 4c are the radial stresses of the wound GDC roll before and after aging. Figure 4d presents the variation of the measured radial stresses for case 2, where the radius ratios are 1.10, 1.35, and 1.85, according to the aging time.

As shown in Figure 4b,c, the measured radial stresses are higher than the estimated radial stresses by 38.7%, 38.9%, and 27.1% before aging when the constant tension, linear taper, and parabolic taper tension profiles were applied, respectively. This change might be because coated parts tend to protrude in the wound layer rather than in the layer before winding. The radial stress can be concentrated in protruding parts [20]. As shown in Figure 4b,c, the radial stress increases with decreasing radius ratio, and the radial stress is close to zero at the maximum radius ratio. The radial stress values expressed in Figure 4c can be specifically confirmed in Table 3. The radial stress in the constant tension is the highest, followed by the linear and then the parabolic taper tension profiles. The parabolic taper tension profile decreases the radial stress more effectively than the linear taper tension profile. This is because the decrement rate of the winding stress in the parabolic taper tension profile, at the beginning of the winding process, is much larger than that in the linear taper tension profile. Moreover, as shown in Figure 4d, the radial stress at the inner part converges more slowly than the radial stress at the outer part. The theoretical and experimental results were used to analyze the defects in the wound GDC layer, depending on the taper tension profile and aging time.

## 4. Defects in the Coated Layer

**Figure 5:**
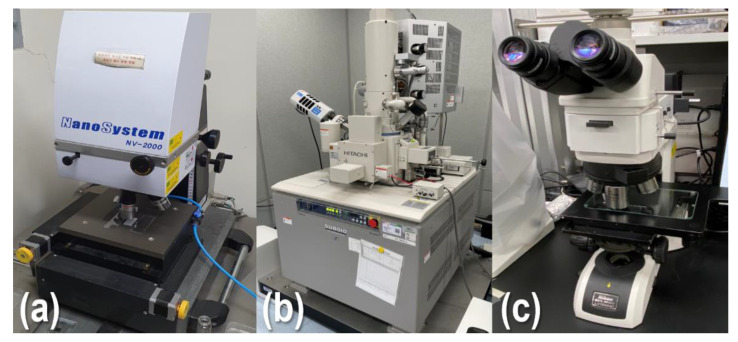
Coated layer measurement system: (**a**) interferometer; (**b**) scanning electron microscope (SEM); and (**c**) polarizing microscope.

The surface and cross-section of the nanoparticle-based GDC-layer wound at radius ratios of 1.10, 1.35, 1.85, and 2.45 before and after aging, respectively, were observed to analyze the defects in the GDC-coated layer due to the radial stress. The coated layer samples were measured using a polarizing microscope (Eclipse LV100ND, Nikon, Tokyo, Japan), an interferometer (NV-2000, NanoSystem Co. Ltd., Daejeon, Korea), and a scanning electron microscope (SEM, SU8010, Hitachi Inc., Tokyo, Japan), respectively. Figure 5a–c present the interferometer, SEM, and microscope used in this study. The thickness of the coated layer was measured seven times, and the five measured thicknesses (after exclusion of the maximum and minimum values) were used to analyze the thickness change of the coated layer, according to the radius ratio of the wound roll.

**Figure 6:**
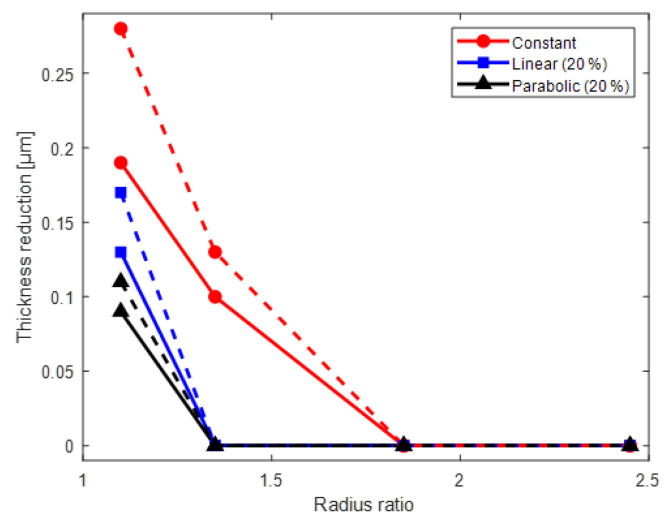
Thickness reduction with respect to the winding tension and aging time.

Figure 6 presents the variation in the thickness according to the radius ratio, taper tension profile, and aging time. In Figure 6, the thickness variation is expressed as the ratio of the decrease in the thickness of the coated layer by the radial stress to the initial thickness without the radial stress. The decrease in the thickness of the wound coated layer is more at the wound layer that is closer to the core. The bold and dotted lines in Figure 6 show the thickness variation before and after aging depending on the winding tension profile. The decrease in the thickness after aging is largest under constant tension (case 2).

**Figure 7:**
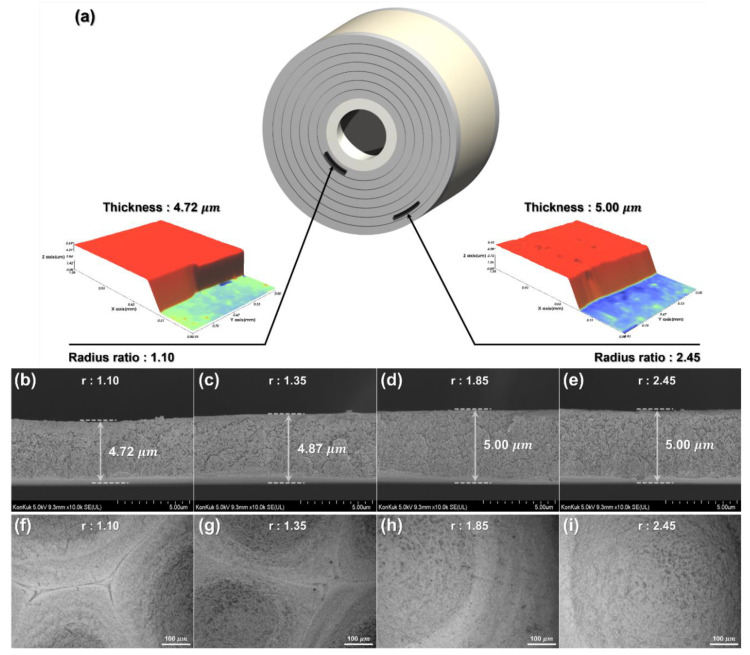
GDC-coated layer in the wound roll for case 2: (**a**) GDC-coated layers measured with the interferometer; (**b**–**e**) cross-sectional images of the GDC-coated layer located at radius ratios of 1.10, 1.35, 1.85, and 2.45, respectively; (**f**–**i**) surface images of the GDC-coated layer located at radius ratios of 1.10, 1.35, 1.85, and 2.45, respectively.

Figure 7a shows a schematic diagram of the wound GDC layer in case 2, which has the most severe thickness reduction defect. GDC-coated layer samples were produced after the unwinding process, and the thickness of the sample was observed with the interferometer shown in Figure 5a. A thickness reduction defect was noticeable at the radius ratio of 1.10, which is close to the center core, and the defect did not occur at the radius ratio of 2.45. Figure 7b–e present the cross-sectional image of the coated layer in case 2 with radius ratios of 1.10, 1.35, 1.85, and 2.45, taken with the SEM shown in Figure 5b. The coated layer thicknesses wound at the layers with radius ratios of 1.10 and 1.35 were decreased by 5.60% and 2.60%, respectively, compared to the coated layer with a radius ratio of 2.45. The thickness of the coated layer located at the radius ratio of 1.85 and 2.45 remained unchanged. In the linear taper profile and parabolic taper profile (cases 3–6), the coated layer thickness decreased only for the layer with a radius ratio of 1.10. The thickness decreased when the radial stress is larger than the yield strength of the GDC-coated layer, and this deformation did not occur at a point where it was less than the yield strength. The thickness decrement of the coated layer increased significantly at the point of the wound roll closer to the core. Particularly, in the winding process, the radial stress increased with the residual stress inside the wound roll, according to the aging time, and thus the thickness reduced further. For a constant tension, after 16 h of aging, the thickness decrement was increased by 1.78%, while it was increased by 0.80% for the linear profile, and 0.40% for the parabolic profile, when compared to the thickness decrement immediately after the winding process.

If the thickness of the GDC electrolyte layer manufactured by roll-to-roll slot-die coating is changed by radial stress as described above, then the geometry of the electrolyte layer might be different from that of a predesigned SOFC and thus cannot be used. In addition, according to Khan’s study, the durability of the SOFC cathode may vary depending on the thickness of the GDC-coated layer, so it is important to maintain the uniformity of the thickness of the GDC-coated layer stored in a wound roll form [28]. Figure 7f–i show the surface images taken with the microscope shown in Figure 5c. The surfaces with radius ratios of 1.10, 1.35, 1.85, and 2.45 of the case 2 wound roll are shown. The wound-coated layers with radius ratios of 1.10 and 1.35 show cracks on the surface in addition to the thickness reduction defect due to the excessive radial stress. The crack was not observed at the relatively outer layers (with radius ratios of 1.85 and 2.45). Moreover, the crack started to occur at a radius ratio greater than 1.35, and the crack was the most severe at a radius ratio of 1.10. The SOFCs manufactured from electrolytes with cracks have a significantly degraded performance, so it is important to prevent such coated layer defects in advance to maintain performance [29].

**Figure 8:**
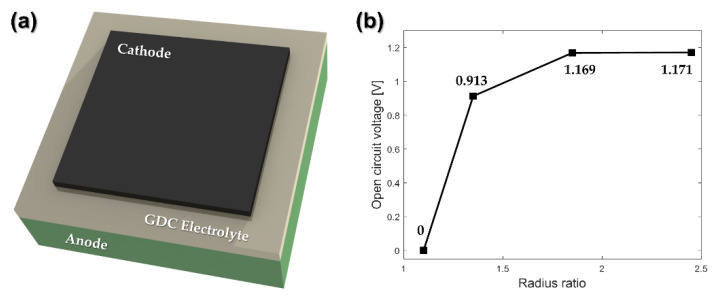
(**a**) Schematic diagram for the fabricated GDC-based SOFC structure; (**b**) open circuit voltage (OCV) for case 2, according to the radius ratio.

As shown in Figure 8a, we manufactured SOFCs to compare the electrical performance according to the coated layer defects using the GDC electrolyte in the wound roll for case 2. We measured the open circuit voltage (OCV), an indicator commonly used for evaluating the performance of the SOFC electrolyte layer [30]. The OCV values for all samples were measured at 700 °C using a potentiostat, with typical SOFC test conditions applied. Figure 8b displays the OCV measurement value depending on the radius ratio in the wound roll. As it is located closer to the center of the wound roll, the OCV of the GDC electrolyte layer tends to decrease. The OCV value of the SOFC manufactured from the GDC coated layer located at a radius ratio of 1.10 could not be measured, which is caused by a crack defect occurring in the coated layer. As the thickness of the electrolyte layer decreases due to excessive radial stress inside the wound roll, the oxygen permeation flux increases [31]. In Figure 8b, the OCV of the GDC-coated layer located at a radius ratio of 1.35 is measured to be lower than that of the outer coated layer, since the oxygen permeation flux and the OCV are inversely proportional.

Therefore, the thickness reduction of the coated layer in the machine direction and the crack on the surface may cause a difference in the performance of the final product. Because these performance differences are related to the yield of the electrolyte layer, it is essential to minimize these defects in roll-to-roll manufacturing system-based processes. Accordingly, it is essential to select a desirable taper tension profile to reduce the radial stress and residual stress in the wound roll in advance to prevent such coated layer defects. In addition, the winding process should be performed in consideration of the permissible range of radial stress, and the storage time of the wound product should be minimized to reduce the effect of the residual stress.

## 5. Conclusions

This study analyzed the effect of radial stress, which increases over time due to residual stress, in the process of winding a nanoparticle-based GDC-coated layer using a roll-to-roll slot-die coating system. According to Burns’ model, the radial stress, hoop stress, and residual stress are generated in the wound roll, and the winding tension is the key factor that determines internal stresses. To analyze radial stresses according to the winding tension profile, constant tension, linear taper tension, and parabolic taper tension profiles were applied. The radial stress was measured with respect to the aging time for each winding tension profile. The radial stress was highest in the constant tension, followed by the linear and parabolic taper profiles, and the increment of the radial stress, according to the aging time, was also in the same order. In this study, we confirmed thickness reduction of the coated layer and crack defects on the surface caused by radial stress and residual stress. The results suggest that even if a manufacturer fabricates a coated layer with a uniform thickness through perfect coating and drying processes, the final product might not be uniform in machine direction (MD) and have cracks on the coated surface in the case of excessively high radial stress. To improve these defects, appropriate taper tension profiles and taper values should be selected to reduce the radial and residual stresses in the wound roll. In addition, we suggest performing the winding process in consideration to the permissible range of radial stresses in advance and storing the wound product for a minimal period to reduce the effect of residual stress.

In this study, a parabolic tension profile with a taper value of 20% was applied to improve the thickness reduction defects and surface crack defects. As a result, cracks did not occur on the surface of the coated layer. The thickness uniformity of the coated layer in MD was 98.2%, and the effect of the residual stress was also reduced, so the thickness uniformity was 97.8%, even after 16 h of aging. The results indicated that the parabolic taper profile is useful for minimizing thickness decrease and the surface cracks of the GDC-coated layer. This study can be used to determine a desirable taper tension profile and taper value, with consideration to the storage time, to minimize performance differences in the electrolyte layer with a roll-to-roll manufacturing system.

## Figures and Tables

**Table 1:** Properties of the PET film, gadolinium-doped ceria (GDC) ink, and GDC-coated layer.

Material	Property	Unit	Value
PET film	Width	mm	150
	Thickness	mm	0.1
	Density	kg/m^3^	1390
	Elastic modulus	MPa	2500
	Poisson’s ratio	-	0.33
	Yield strength	MPa	110
GDC ink	Particle size	nm	<500
	Viscosity	cP	85
	Solid contents	wt%	44
	Contact angle	°	35
	Surface tension	mN/m	32
GDC-coated layer	Width	mm	120
	Density	kg/m^3^	6700
	Elastic modulus	MPa	3100
	Poisson’s ratio	-	0.28
	Yield strength	MPa	1.95

**Table 2:** Coating and winding process conditions for cases 1–6.

Conditions	Unit	Case 1	Case 2	Case 3	Case 4	Case 5	Case 6
Coating gap	mm	0.1	0.1	0.1	0.1	0.1	0.1
Flow rate	mL/min	6.7	6.7	6.7	6.7	6.7	6.7
Drying temperature	°C	45	45	45	45	45	45
Web speed	m/min	1	1	1	1	1	1
Initial winding tension	kgf	2.7	2.7	2.7	2.7	2.7	2.7
Taper tension profile	-	Constant	Constant	Linear	Linear	Parabolic	Parabolic
Taper value	%	-	-	20	20	20	20
Maximum radius ratio	-	2.50	2.50	2.50	2.50	2.50	2.50
Aging time	hour	0	16	0	16	0	16

**Table 3:** Measured radial stress depending on the taper tension profile and aging time.

Radius Ratio	Radial Stress [MPa]					
	Case 1	Case 2	Case 3	Case 4	Case 5	Case 6
1.10	2.752	3.358	2.264	2.604	1.995	2.196
1.35	2.046	2.308	1.616	1.758	1.417	1.502
1.85	0.991	1.052	0.725	0.756	0.697	0.713
2.50	0	0	0	0	0	0

## Data Availability

The data presented in this study are available from the corresponding author upon reasonable request.
